# Sensitive and rapid quantification of exosomes by fusing luciferase to exosome marker proteins

**DOI:** 10.1038/s41598-018-32535-7

**Published:** 2018-09-19

**Authors:** Tomoya Hikita, Mamiko Miyata, Risayo Watanabe, Chitose Oneyama

**Affiliations:** 1 Division of Cancer Cell Regulation, Aichi Cancer Center Research Institute, Chikusa-ku, Nagoya, Japan; 2 JST, PRESTO, Nagoya, Japan

## Abstract

Exosomes have emerged as important mediators of intercellular communication. Although their modes of action have been elucidated, the molecular mechanisms underlying their secretion, sorting of molecules, uptake into recipient cells, and biological distribution *in vivo* remain elusive. Here, we present a novel system for quantifying secreted exosomes by introducing ectopic or CRISPR/Cas9-mediated knock-in of luciferase-fusion exosome markers such as CD63. This luciferase-based method makes it possible to measure exosomes secreted into the culture medium with high linearity and wide dynamic range in a high-throughput manner. We demonstrate that data obtained by luminescent quantification are well correlated with data obtained by conventional nanoparticle tracking analysis under multiple conditions. In addition, our system is capable of evaluating the recipient cells or tissues that take up exosomes, as well as visualizing exosomes *in vivo*. The proposed system represents a powerful tool for understanding the molecular mechanisms underlying exosome production, uptake, and long-term distribution.

## Introduction

Exosomes, 50–150-nm extracellular vesicles secreted by various types of cells, contain cell-derived biological molecules such as nucleic acids (DNA, mRNA, lncRNA, and miRNA), proteins, and lipids. Secreted exosomes are taken up into proximally or distally located cells and modulate biological functions within recipient cells^1^. Therefore, exosomes have recently attracted attention as an intercellular communication system. Under normal physiological conditions, they play pivotal roles in the regulation of stem cell maintenance^2,3^, tissue repair^4^, and immuno-surveillance^5,6^. In addition, exosomes are involved in cancer progression by preparing the tumor microenvironment and pre-metastatic niche.

Cancer cells secrete aberrantly large amounts of exosomes that contain different cargo molecules than those from normal cells, depending on malignant cancer phenotypes^7–10^; that is, cancer cells progress by altering the exosome quality (cargo variety) and quantity (level of production). Based on these differences between normal and cancer cells, liquid biopsies using exosomes for cancer diagnosis have advanced rapidly^11–13^. In addition, therapeutic strategies targeting cancer-derived exosomes would also be useful^14^. Although methods for removing circulating cancer-derived exosomes by extracorporeal hemofiltration or antibodies are currently under investigation^15,16^, to date no therapeutic medicines or strategies targeting exosome secretion have been developed^17^. This is largely because the lack of a high-throughput system for measuring exosomes makes it hard to elucidate the molecular mechanisms underlying their secretion or to test candidate inhibitors. Hence, high-throughput and high-precision exosome quantification systems will be indispensable for the development of clinical and biological exosome studies in the future.

Previously, various physico- or bio-chemical methods, strategies, and devices have been developed and adapted for the assessment of exosome quantity. Among them, ultracentrifugation (UC)-based methods have been commonly used in combination with subsequent quantification by nanoparticle tracking analysis (NTA) or immunoblotting. These conventional methods are useful for analyzing the amount of exosomes, but UC-based methods are time-consuming and their exosome recovery rates are low^18^. Although NTA enables one to quantitatively analyze not only the concentration but also the size distribution of exosomes, it does not allow measurement of multiple samples simultaneously, and comparisons of values obtained with different instrument settings and conditions must be performed with caution^19^. Biochemical techniques such as ELISA, AlphaLISA, and flow cytometry (FCM) also have been utilized for exosome quantification^8,20–23^. However, while these methods enable one to quantify specific populations of exosomes in both culture medium and body fluids without isolation, they require time-consuming pre-treatment of target samples or optimization of measurement conditions. In addition, although microfluidic devices such as ExoChip or nPLEX capable of quantifying exosomes from small amounts of sample without isolation have been recently developed^24–26^, they remain commercially unavailable or very expensive.

Given this situation, a sensitive, rapid, easy, and low-cost technique for exosome quantification is desired. In this study, we developed a cell-based high-throughput exosome quantification system by genetically labeling exosome markers such as CD63, CD9, and CD81 with high-intensity luciferase NanoLuc (Nluc)^27^. Using this system, the intensity of luciferase luminescence was well correlated with the number of exosomes in the cell culture medium. Furthermore, Nluc-labeled exosome-secreting cells and/or secreted exosomes could also be used to evaluate recipient cells that take up exosomes and to visualize the distribution of intact exosomes *in vivo*. Taken together, our results demonstrate that the novel luciferase–exosome system could be an effective tool for analyzing the molecular mechanisms underlying secretion, uptake, and long-term spatial behavior of exosomes.

## Results

### Quantification of exosomes with Nluc-fused CD63

**Figure 1:**
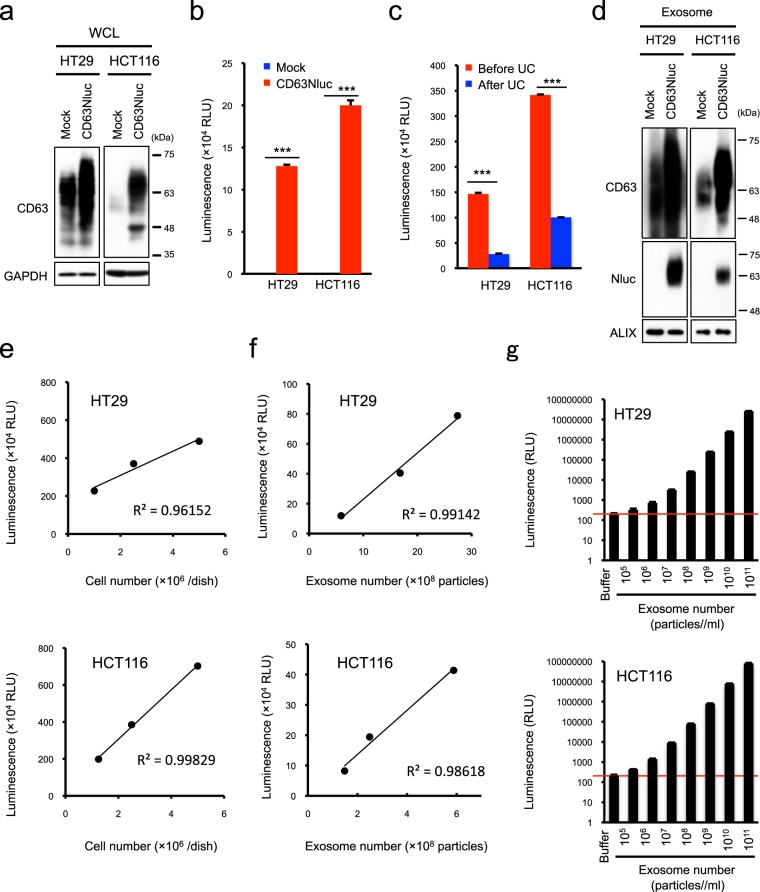
Ectopic Nluc-fused CD63 expression enables quantification of exosome production. (**a**) Western blot analysis of CD63 expression. (**b**) NanoLuc luciferase intensity in the culture medium of control (Mock) and Nluc-fused CD63-expressing HT29 and HCT116 cells (CD63Nluc). (**c**) Nluc intensity in the culture medium before and after ultracentrifugation (UC). (**d**) Western blot analysis of CD63 expression in exosomes secreted from control (Mock) and Nluc-fused CD63-expressing cells (CD63Nluc). ALIX was used as an exosome marker protein. (**e**) Correlation between luciferase intensity (in the culture medium) and cell number. The solid line shows the linearity of the fitted curve of luminescence vs. seeded cell number. (**f**) Correlation between luciferase intensity and exosome number. The solid line shows the linearity of the fitted curve luminescence vs. exosome number. (**g**) Detection limits of CD63Nluc-expressing HT29 and HCT116 cells in exosome quantification. Purified exosomes were adjusted to a concentration of 10^11^ particles/mL, and a dilution series was prepared down to a concentration of 10^5^ particles/mL. Detection limits were decided by comparing luciferase intensities of the dilution series with those of buffer (20 mM HEPES, pH7.4). The number of purified exosomes was measured by NanoSight, and luminescence was measured by a luminometer. Results are expressed as means ± SD of three wells. All data are representative of at least three independent experiments. ***P < 0.001 by two-tailed Student’s *t*-test. Uncropped gel images for panels a and d are shown in Supplementary Fig. 6.

Tetraspanin CD63 has recently been used as a representative exosomal marker protein. To quantify cell-released exosomes by luminescence, we first attempted to label exosomes with Nluc by ectopically expressing Nluc-fused CD63 in HT29 and HCT116 cells (Fig. 1a). Luciferase activity was easily detectable in the culture medium of CD63Nluc-expressing cells, but not control cells (Fig. 1b). To determine whether the luminescence in the culture medium was derived from exosomes, we compared the luminescence intensities before and after ultracentrifugation and quantified CD63Nluc protein in isolated exosomes. Ultracentrifugation drastically reduced the intensity of luminescence in the culture medium (Fig. 1c), and CD63Nluc was detected only in exosomes derived from CD63Nluc-expressing cells (Fig. 1d and Supplementary Fig. 1). These data indicate that ectopically expressed CD63Nluc can label exosomes with Nluc. To determine whether the luminescence intensity of CD63Nluc-expressing cells provides a quantitative report of exosome number, we next investigated the relationship between reporter signal intensity and cell number or exosome number in the culture medium. When CD63Nluc-expressing cells were seeded at various densities, luminescence in the culture medium correlated closely with both cell and exosome numbers (Fig. 1e,f). Furthermore, we determined the absolute detection limit for exosome quantification using CD63Nluc-expressing cells. At a concentration of 10^5^ particles/mL, the luminescence intensity of Nluc-labeled exosomes was higher than the background level. The regression line depicting the correlation between luminescence and exosome number was linear, in a statistically significant manner, at concentrations above 10^6^ particles/mL (Fig. 1g). Furthermore, we investigated whether ectopic CD63Nluc expression influenced biological characteristics such as cell growth and exosome production. CD63Nluc was found to be localized in multivesicular bodies (MVBs) as endogenous CD63 (Supplementary Fig. 1). Ectopic expression of CD63Nluc did not show significant effects on cell growth and the number and size of exosomes secreted from CD63Nluc-expressing cells were almost equivalent to those from control cells (Supplementary Fig. 1). Taken together, these data suggest that CD63Nluc-expressing cells represent a useful tool for directly quantifying exosomes without purification.

### Reliability of CD63Nluc-expressing cells in exosome quantification

**Figure 2:**
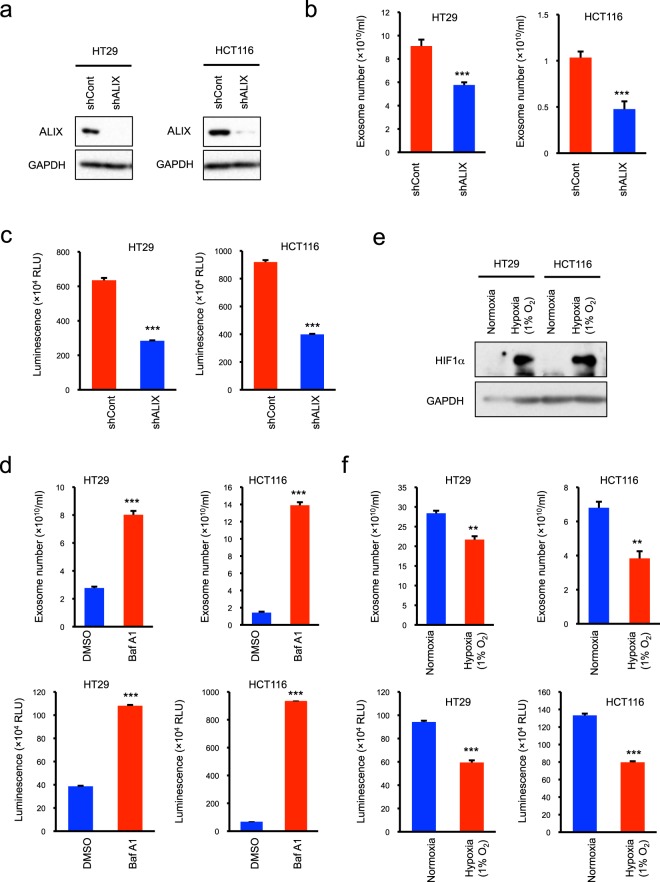
Luciferase activity in culture medium exactly reflects exosome production. (**a**) Western blot analysis of ALIX expression. Total cell lysates from CD63Nluc-expressing cells treated with control (shCont) or ALIX shRNA (shALIX) were immunoblotted with anti-ALIX and anti-GAPDH antibodies. (**b**) Exosome numbers produced from control and ALIX-suppressed CD63Nluc-expressing HT29 (left panel) and HCT116 (right panel) cells. (**c**) Luminescence in the culture medium of control and ALIX-suppressed CD63Nluc-expressing HT29 (left panel) and HCT116 (right panel) cells. (**d**) Exosome number produced by CD63Nluc-expressing HT29 and HCT116 cells treated with DMSO or 50 nM bafilomycin A1 (BafA1) (lower panels). Luminescence in the culture medium from the same cells (upper panels). (**e**) Western blot analysis of HIF1α (Hypoxia). Total cell lysates from CD63Nluc-expressing cells cultured for 48 hours under normoxic (20% O_2_) or hypoxic conditions (1% O_2_) were immunoblotted with anti-HIF1α and anti-GAPDH antibodies. (**f**) Exosome number produced from CD63Nluc-expressing HT29 and HCT116 cells cultured for 48 hours under normoxic (20% O_2_) or hypoxic conditions (1% O_2_) (upper panels). Luminescence in the culture medium of the same cells (lower panels). Results are expressed as means ± SD of three wells. All data are representative of at least three-independent experiments. **P < 0.01 and ***P < 0.001 by two-tailed Student’s *t*-test. Uncropped gel images for panels a and e are shown in Supplementary Fig. 6.

For bioluminescent quantification of exosome number, luciferase activity should reflect alterations of exosome production with a high degree of accuracy. To determine the quantitative accuracy of CD63Nluc-expressing cells, we artificially controlled exosome production by genetic and pharmacological techniques. Because ALIX, an ESCRT-associated protein, participates in the formation of intraluminal vesicles (ILVs) at multivesicular bodies (MVBs)^28,29^, we first investigated whether CD63Nluc-expressing cells are applicable to detect ALIX-mediated exosome production (Fig. 2a). Nanotracking analysis (NTA) revealed that shRNA-mediated suppression of ALIX expression significantly decreased exosome production (Fig. 2b). Consistent with this, the luminescence in the culture medium was significantly lower in ALIX-suppressed cells than in control cells (Fig. 2c).

We next investigated whether CD63Nluc-expressing cells are useful to detect alteration of exosome biogenesis following drug treatment. Several groups have shown that bafilomycin A1, a V-ATPase inhibitor, enhances exosome production^30–33^. NTA confirmed that bafilomycin A1 prominently increased exosome production (Fig. 2d). Interestingly, luminescence in the culture medium increased at the same rate as the exosome number (Fig. 2d). In addition to these genetic and pharmacological effects, several environmental factors are also critical for exosome biogenesis and release. Here, we studied the relationship between bioluminescence in the culture medium and secreted exosome number in a hypoxic culture. NTA showed that exosome number decreased under hypoxia, when HIF1α was induced (Fig. 2e,f). Although the hypoxic effect on exosome secretion is dependent on cell types and experimental conditions in previous reports^34–37^, it is noteworthy that luminescence in the culture medium also decreased along with exosome number in this study (Fig. 2f). Collectively, these results indicate that CD63Nluc-expressing cells enable exact quantification of exosome production, as well as alteration of production by a variety of factors.

### Quantification of exosomes in Nluc-labeled CD9 and CD81

**Figure 3:**
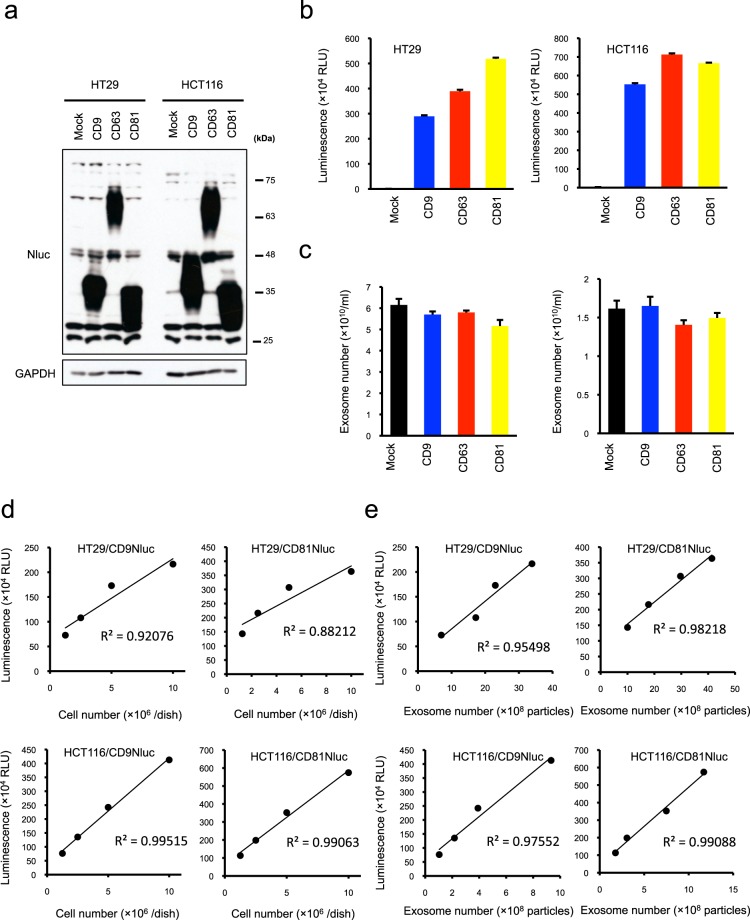
CD9 and CD81 are also useful for quantifying exosome production. (**a**) Western blot analysis of ectopic CD9, CD63, and CD81 expression. Total cell lysates from CD9, CD63, and CD81Nluc-expressing HT29 and HCT116 cells were immunoblotted with anti-Nluc and anti-GAPDH antibodies. (**b**) NanoLuc luciferase intensity in the culture medium of control (Mock) and CD9, CD63, or CD81Nluc-expressing HT29 and HCT116 cells. (**c**) Exosome number in the culture medium of control (Mock) and CD9, CD63, or CD81Nluc-expressing HT29 (left panels) and HCT116 cells (right panels). (**d**) Correlation between luciferase intensity (in the culture medium) and cell number. The solid line shows the correlation between luminescence and seeded cell number. (**e**) Correlation between luciferase intensity and number of exosomes. The number of purified exosomes was measured by NanoSight, and the luminescence was measured on a luminometer. Results are expressed as means ± SD of three wells. All data are representative of at least three-independent experiments. Uncropped gel images for panel a are shown in Supplementary Fig. 6.

Because exosomes consist of subpopulations that represent distinct biological characteristics and sizes^38^, it is necessary to select a proper marker protein for labeling exosomes with Nluc according to the target nanoparticle subset. Therefore, we evaluated whether exosome markers other than CD63, such as CD9 and CD81, could be utilized in Nluc-mediated exosome quantification. To achieve this end, we developed Nluc-fused CD9- and CD81-expressing cells (Fig. 3a). As with CD63Nluc-expressing cells, both CD9Nluc and CD81Nluc-expressing cells exhibited very high luciferase intensities (Fig. 3b). On the other hand, ectopic CD9Nluc or CD81Nluc expression did not show significant effects on the production of exosomes in the parental cells (Fig. 3c). As before, we confirmed that the luminescence was derived from exosomes by comparing luminescence intensities before and after ultracentrifugation (Supplementary Fig. 2). Furthermore, to determine whether CD9- and CD81Nluc-expressing cells quantitatively reflected exosome number as luminescence intensity, we investigated the correlation between reporter signal intensity and cell or exosome number in the culture medium. When CD9- or CD81Nluc-expressing cells were seeded at various cell numbers, the luminescence of the culture medium was closely correlated with the cell and exosome number (Fig. 3d,e). These data indicate that CD9 and CD81 can also be utilized as effective markers for quantifying exosomes in the Nluc-based detection system.

### Generation of exosome-detectable cells by knocking Nluc into endogenous CD63

**Figure 4:**
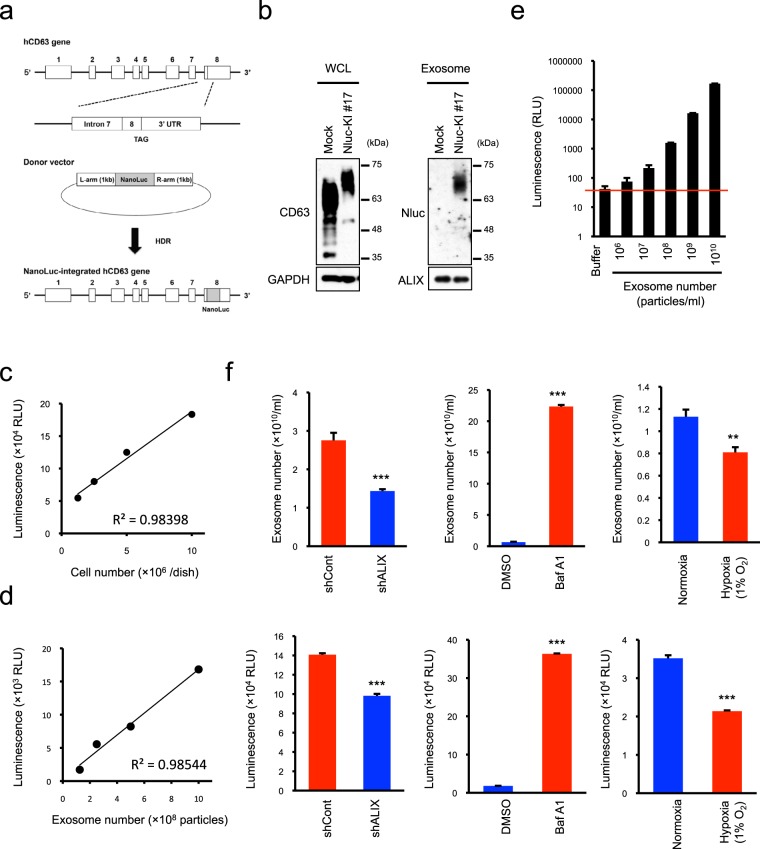
Generation of CD63Nluc-knock-in-HCT116 cells. (**a**) Schematic representation for generating CD63Nluc knock-in-HCT116 cells. (**b**) Western blot analysis of Nluc-labeled intrinsic CD63 expression in cells (left panels) and purified exosomes (right panels). ALIX was used as an exosomal marker protein. (**c**) Correlation between luciferase intensity (in the culture medium) and cell number. The solid line shows the linearity of the fitted curve between luminescence and seeded cell number. (**d**) Correlation between luciferase intensity (in the culture medium) and exosome number. Solid line shows the linearity of the fitted curve of luminescence vs. exosome number. (**e**) Detection limits of CD63Nluc-KI#17-HCT116 cells for exosome quantification. Purified exosomes were adjusted to a concentration of 10^10^ particles/mL, and then a dilution series was prepared down to a concentration of 10^6^ particles/mL. Detection limits were determined by comparing luciferase intensities of the dilution series with those of buffer (20 mM HEPES, pH7.4). (**f**) Alteration of exosome number (upper panels) and luminescence (lower panels) in the culture medium following treatment of CD63Nluc-KI#17-HCT116 cells with ALIX shRNA (left panels), bafilomycin A1 (middle panels), or hypoxia (1% O_2_) (right panels). Results are expressed as means ± SD of three wells. All data are representative of at least three-independent experiments. **P < 0.01 and ***P < 0.001 by two-tailed Student’s *t*-test. Uncropped gel images for panel b are shown in Supplementary Fig. 6.

Although Nluc-fused CD63 expression was useful for quantifying exosomes with high sensitivity and high accuracy (Fig. 1) and did not show significant effects on cell growth or exosome production in HT29 and HCT116 cells (Supplementary Fig. 1), the tetraspanin CD63 is associated with multiple biological functions in addition to exosome production^39,40^. Therefore, it would desirable to avoid overexpression of CD63 when monitoring alterations in the rate of intrinsic exosome production. To this end, we next attempted to directly label the *CD63* gene with Nluc using the CRISPR/Cas9 genome-editing system. To insert the Nluc gene sequence upstream of the 3′ terminal stop codon, we constructed a targeting vector and knock-in donor vector, and co-transfected both vectors into HCT116 cells (Fig. 4a). We selected some candidate clones by using luciferase activity as an indicator of Nluc knock-in, and obtained CD63Nluc knock-in (KI) cells (clone#17) after confirming the introduction of Nluc by PCR (Supplementary Fig. 3). Finally, we sequenced the *CD63* gene in this clone and confirmed homozygotic Nluc insertion at the preterminal position (Supplementary Fig. 3). Expression of Nluc-labeled CD63 was detected in whole cells and isolated exosomes only in CD63Nluc-KI #17 cells (Fig. 4b). Nluc knock-in did not show significant effects on the localization of CD63 and the number and size of exosomes (Supplementary Fig. 3). As described above for CD63Nluc-expressing cells, we studied the relationship between reporter signal intensity and cell number or exosome number in the culture medium. Reporter signals in the culture medium were closely correlated with both cell and exosome numbers (Fig. 4c,d). Moreover, the curve depicting the correlation between luminescence and exosome number was linear in a statistically significant manner at concentrations above 10^6^ particles/mL (Fig. 4e). Furthermore, to verify the reliability of CD63Nluc-KI #17 for exosome quantification, we monitored the alterations of exosome number and luminescence in the culture medium from cells treated with ALIX shRNA, bafilomycin A1, and hypoxia. Under all conditions, changes in the luminescence of the culture medium reflected the alterations in the exosome number (Fig. 4f). Taken together, these results suggest that knock-in of Nluc into CD63 provides a useful tool for quantifying exosomes.

### Application of Nluc-labeled exosome–producing cells to cellular uptake analysis

**Figure 5:**
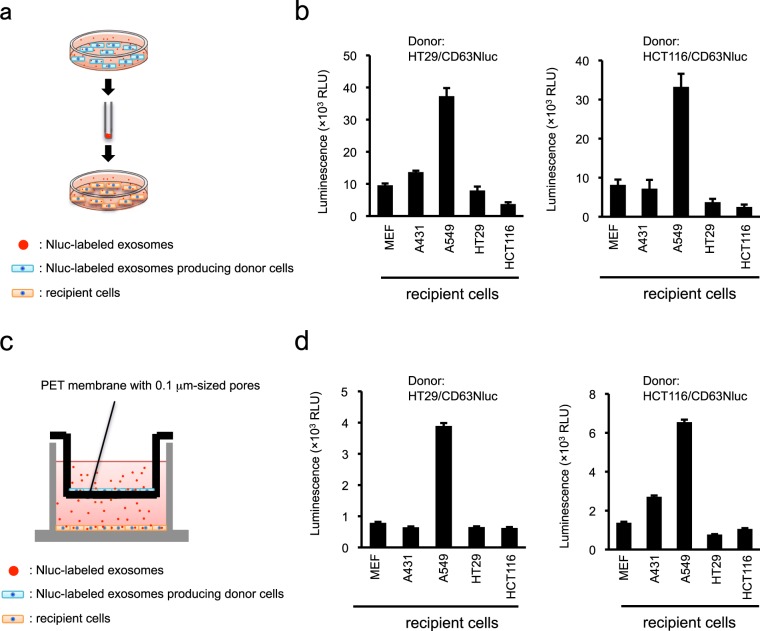
Application of CD63Nluc-expressing cells to the exosome uptake analysis. (**a**) Schematic representation for analyzing uptake efficiency of exosomes in a direct addition system. After Nluc-labeled exosomes isolated by ultracentrifugation were added to the recipient cells, internalized Nluc was detected as luminescence. (**b**) Nluc intensity in recipient cells, as determined by uptake assay with direct addition. (**c**) Schematic representation for analyzing uptake efficiency of exosomes in a co-culture system. After recipient cells were cultured with CD63Nluc-expressing cells as the exosomes source, internalized Nluc in recipient cells was detected as luminescence. (**d**) Nluc intensity in recipient cells, as determined by uptake assay in co-culture. Results are expressed as means ± SD of three wells. All data are representative of at least three-independent experiments.

Exosomes work as intercellular mediators only after they are incorporated into recipient cells. Therefore, it is important to quantitatively evaluate the uptake efficiency of exosomes. We speculated that Nluc-labeled exosomes or CD63Nluc-expressing cells would enable quantitative measurement of exosome uptake. First, we added a large amount of exosomes isolated from CD63Nluc-expressing cells to five different types of recipient cells, and measured the luminescence intensities in each cell type (Fig. 5a). Each recipient cell had a different luminescence intensity, with A549 human lung cancer cells exhibiting the most luminescent (Fig. 5b). Uptake assay systems involving the addition of labeled exosomes has been widely used to investigate the cellular uptake of exosomes. However, this system could yield non-physiological results because very high levels of exosome were added. Therefore, we next quantified the uptake efficiency of exosomes by culturing CD63Nluc-expressing cells with recipient cells (Fig. 5c). Although the luminescence intensities in recipient cells were lower overall, the co-culture system yielded a luminescence pattern nearly identical to that of the exosome addition system (Fig. 5d). Taken together, these data suggest that CD63Nluc-expressing cells or Nluc-labeled exosomes are suitable for quantitative analysis of exosome uptake.

### Application of Nluc-labeled exosome–producing cells to long-term biodistribution analysis

**Figure 6:**
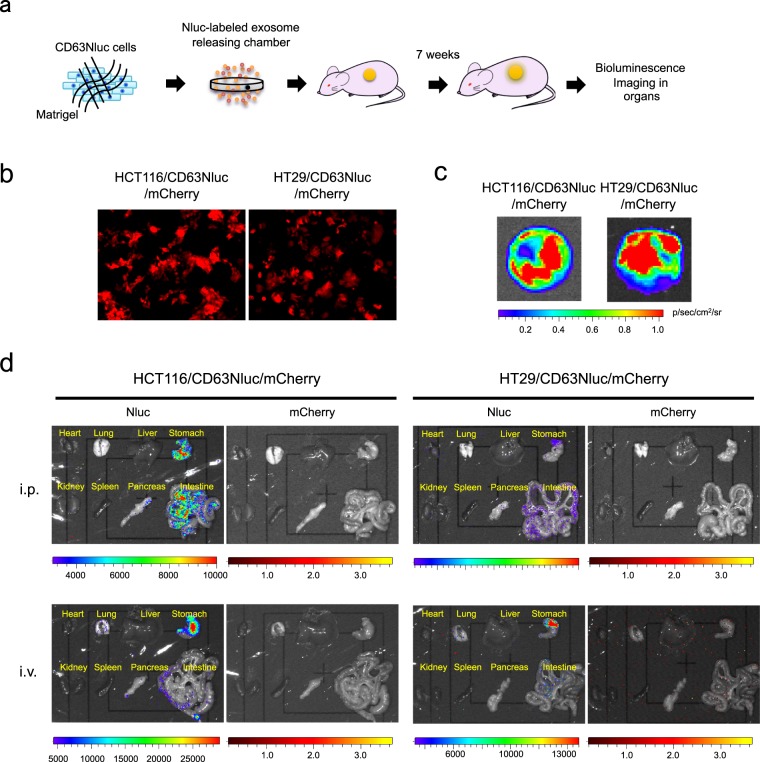
Application of CD63Nluc-expressing cells to long-term biodistribution analysis of exosomes. (**a**) Schematic representation of analysis of the long-term biodistribution of exosomes. Chamber rings loaded with HT29 or HCT116 cells stably expressing CD63Nluc and mCherry (HT29/CD63Nluc/mCherry or HCT116/CD63Nluc/mCherry) and Matrigel were dorsally implanted into 5-week-old Balb/c-nu/nu female mice. At 7 weeks after implantation, furimazine, a Nluc substrate, was intravenously or intraperitoneally administered, and the luminescence in each organ was imaged by IVIS. (**b**) Immunofluorescence analysis in HT29/CD63Nluc/mCherry or HCT116/CD63Nluc/mCherry cells. (**c**) Bioluminescence images in exosome-releasing chamber harvested from mice 7 weeks after implantation. (**d**) Bioluminescence images in organs harvested from exosome-secreting chamber-bearing mice 7 weeks after implantation. Substrate was intraperitoneally (i.p.) or intravenously (i.v.) injected into mice and luciferase activities derived from Nluc were imaged with IVIS.

Luciferase is widely exploited for *in vivo* imaging of cells, proteins, and molecules such as drugs. Therefore, we investigated whether cells secreting CD63Nluc-labeled exosomes are useful for analyzing the biodistribution of exosomes. Exosomes secreted from cells constantly circulate throughout the whole body via the blood. Therefore, we developed an experimental system that persistently releases exosomes *in vivo*: specifically, we subcutaneously implanted CD63Nluc-expressing cells encapsulated in a chamber ring with Matrigel into mice (Fig. 6a). Although the encapsulated cells never physically escape from the chamber ring, we labeled the CD63Nluc-expressing cells with mCherry to distinguish between exosome-derived and cell-derived luminescence (Fig. 6b and Supplementary Fig. 4). At 7 weeks after implantation, we tried to detect exosome-derived luminescence in each organ by intravenously or intraperitoneally injecting furimazine (Fig. 6a). After furimazine injection, the chamber ring emitted strong luminescence (Fig. 6c). These data indicate that the implanted chamber ring consistently supplied Nluc-labeled exosomes to the entire body for 7 weeks. In this persistent circulating exosome model, we observed intense luminescence in the stomach and intestine (Fig. 6d). Because the mCherry signal was not detected in these organs (Fig. 6d), we concluded that the luminescence was derived from homing exosomes. These data indicate that CD63Nluc-expressing cells are suitable for analysis of exosome biodistribution.

## Discussion

Luciferase is the most commonly biolumininescence reporter used for quantitative analysis when monitoring multiple biological phenomena such as promoter activity, cell viability, and protein–protein interactions *in vitro*, as well as tumorigenesis *in vivo*. Although firefly luciferase (Fluc) has traditionally been used in multiple types of biological analyses, smaller and brighter luciferases are also commonly used. *Gaussia* luciferase (Gluc), from the marine copepod *Gaussia princeps*, emits 100-fold higher luminescence in mammalian cells and is much smaller than Fluc (19.9 kDa vs. 61 kDa, making it the better choice for fusion proteins). However, Gluc is naturally secreted and emits a flash-type luminescence, and the signal drops rapidly^41^. By contrast, NanoLuc (Nluc), an engineered luciferase derived from the deep-sea shrimp *Oplophorus*, is a non-secreted protein that exhibits sustained signal duration. In addition, Nluc is 150-fold brighter and more stable under a range of temperatures, pH values, and detergent concentrations. Also, as with Gluc, Nluc is significantly smaller (19 kDa) in size than Fluc or *Renilla* luciferase (36 kDa)^27^. These properties of Nluc make it the most suitable luciferase for labeling of exosomes. Therefore, we developed a cell-based exosome quantification system using Nluc.

Compared to the UC-NTA method, our Nluc-based exosome measurement system has two main disadvantages: it cannot be used to obtain the size distribution of exosomes or to analyze biological samples such as serum or plasma. However, it is superior to the UC-NTA method from the standpoints of measurement sensitivity, accuracy, operability, operating time, throughput, and run cost. The measurement range of Nluc-labeled exosome-producing cells was 10^6^–10^11^ particles/mL, whereas the recommended measurement range of NTA is 10^8^–10^9^ particles/mL^19^ (Figs 1g and 4e). Also, luminescence in the culture medium was linearly correlated with both cell number and exosome number in the range of 10^6^–10^11^ particles/mL (Figs 1e,f, 3d,e and 4c,d). That is, Nluc-labeled exosome–producing cells give a 100-fold wider measurement range than NTA. It is noteworthy that measurements using Nluc-labeled exosome–producing cells do not require exosome isolation by UC, which leads to lower recovery of exosomes in samples^18^. We also investigated the recovery rate of exosomes following ultracentrifugation of samples derived from Nluc-labeled exosome–producing cells. Calculations based on luminescence intensities revealed that the rate of exosome collection by ultracentrifugation was approximately 20% (Supplementary Fig. 5). This implies that most of the exosomes in samples are lost during the ultracentrifugation or washing process. Furthermore, due to its simplified handling and the lack of a requirement for expensive reagents and instruments, exosome quantification using Nluc-labeled exosome–producing cells takes less than 15 minutes for acquisition of data. Overall, the Nluc-based quantification method has several technical advantages: (1) high sensitivity, (2) high accuracy, (3) high reproducibility, (4) ease of handling, (5) speed, and (6) low cost relative to conventional methods. In addition to these advantages, Nluc-based quantification permits detection of exosome-derived luminescence in small-culture scales, e.g., 96- or 384-well format. Therefore, this method would be suitable for high-throughput screening for exploring exosome-associated molecules or exosome-regulated compounds. Because cancer cell-derived exosomes are associated with metastasis, inhibition of exosome production represents a promising therapeutic strategy for cancer^42,43^.

Exosomes are not homogeneous vesicles, but instead consist of a variety of distinct subpopulations depending on size and cargo, including surface proteins, glycosylation, cargo molecules, and lipid composition^44^. Because each subpopulation has distinct biological functions, it is important to consider the heterogeneity of exosomes. Although CD63 is a useful marker for endosome-derived exosomes, some cells secrete subpopulations of exosomes that are devoid of CD63^38,44,45^. In addition, given that CD63 is present in microvesicles, it will be necessary to use other exosome markers in some cases. Therefore, we also investigated whether Nluc-labeling of CD9 or CD81 would enable quantification of exosomes. As with CD63, Nluc-labeled CD9- or CD81-expressing cells reported exosome number as luminescence intensity with high sensitive and accuracy (Fig. 3d,e). CD63Nluc expression was suitable for quantifying the exosomes in HT29 and HCT116 human colon cancer cells. However, we can employ additional adequate exosome markers such as CD9 and CD81 for use in other cell lines and target subpopulations.

Additionally, we should consider the unexpected and unwanted biological effects of overexpression of CD63, CD9, or CD81 in this exosomal quantification system. Although ectopic CD63 expression had no influence on exosome production or cell growth under our experimental conditions, tetraspanins are known to be involved in multiple biological functions^39,40^. Therefore, it would be desirable to conjugate Nluc to an exosome marker without overexpression. Accordingly, we directly inserted the Nluc sequence to the genomic locus of CD63 using CRISPR/Cas9-mediated knock-in. The intrinsic expression level of knock-in Nluc-labeled CD63 was sufficient for exosome quantification based on luminescence, although the sensitivity was insufficient to perform exosome uptake and biodistribution assay. Recently, a screen of a randomly mutated library identified the Nluc mutant ‘teLuc,’ which is about 6-fold brighter than the original Nluc^46^. If further development or improvement of luciferase or substrate enables detection of the weaker luminescence, the knock-in–mediated luciferase labeling strategy would permit not only quantification of exosome number but also the quantitative elucidation of exosome uptake or long-term biodistribution.

In addition to its function as counting devices, CD63Nluc-labeled exosome–producing cells and CD63Nluc-labeled exosomes could be used to evaluate the uptake efficiency of recipient cells or their biodistribution. Here, we quantitatively scored uptake efficiency by adding isolated Nluc-labeled exosomes to recipient cells or by co-culturing Nluc-labeled exosome–producing cells with recipient cells (Fig. 5). Until now, almost all exosome uptake assays have been performed by counting or measuring internalized labeled exosomes under fluorescence microscopy^47^, following conjugation of lipophilic dyes to isolated exosomes. However, lipophilic dyes such as PKH26, PKH67, and DiI, which label lipid-containing entities other than exosomes, produce false-positive signals. Additionally, conjugation of lipophilic dyes potentially disrupts the function of exosome surface proteins^47,48^ that are responsible for binding and subsequent uptake of exosomes. By contrast, cellular uptake assays using CD63Nluc-labeled exosome–producing cells or CD63Nluc-labeled exosomes accurately quantify the uptake efficiency of various recipient cells without requiring exosome isolation and/or labeling. Therefore, the Nluc-based assay would be more appropriate than conventional methods for quantitative assessment of exosome uptake.

We also used CD63Nluc-labeled exosome–producing cells to investigate the biodistribution of exosomes. Although exosomes have emerged as promising new biomarkers for diagnosis and prognosis of cancer, their potential use as drug delivery vehicles have also attracted attention over the years^49^. To monitor the short-term biodistribution of exosomes as drug carriers, a common method is injection of lipophilic dye-conjugated or genetically fluorescence-labeled exosomes into living animals^50^. This analytical approach may be reasonable for elucidating the *in vivo* dynamics of exosomal medicines, but we should not apply the resultant data to the dynamics of exosomes secreted from cells. This is because almost all cells, including cancer cells, persistently release exosomes into the extracellular space, and secreted exosomes constantly circulate through the body via the bloodstream. To improve the exosome supply system, we developed a novel exosome-releasing device by encapsulating CD63Nluc-labeled exosome producer cells in a chamber ring. Implantation of this exosome-releasing device revealed that HT29- and HCT116-derived exosomes preferentially accumulated in the stomach and intestine after a long period of time. Notably in this regard, we previously showed that Src-transformed mouse embryonic fibroblast (MEF)-derived exosomes are exclusively recruited into the lungs (Supplementary Fig. 4). In those experiments, we labeled MEF-derived exosomes expressing Src-Nluc but not CD63-Nluc, so these results are incomparable. However, the Nluc-labeled exosome–releasing chamber is a promising tool for analyzing exosome organotropism in living animals.

In summary, we showed here that Nluc-labeled exosome–producing cells represent a powerful approach for evaluating exosome production, uptake, and long-term tissue disposition. This system is very easy to establish, requiring only basic molecular biological techniques. We hope that this quantification method will be widely adopted, and that it will aid in the advancement of exosome biology.

## Methods

### Ethics approval

All animal experiments were performed under protocols approved by the Animal Care and Use Committee of Aichi Cancer Center Research Institute. All analyses were performed in accordance with approved guidelines and regulations.

### Cell culture and regents

Human colon cancer cell lines HCT116, HT29 cells and human lung cancer cell lines A431, A549 were obtained from American Type Culture Collection (ATCC). Mouse embryonic fibroblast (MEF) immortalized by large-T antigen was kindly provided from Dr Akira Imamoto^51^. HCT116, HT29 and generated CD63Nluc-KI/HCT116 cells were cultured at 37 °C with 5% CO_2_ in McCoy’s 5A medium (Thermo Fisher Scientific, Waltham, MA, USA) supplemented with 10% fetal bovine serum (Thermo Fisher Scientific). A431, A549 and MEF were cultured at 37 °C with 5% CO_2_ in Dulbecco’s Modified Eagle’s Medium (DMEM: Sigma-Aldrich, St Louis, MO, USA) supplemented with 10% fetal bovine serum (Thermo Fisher Scientific). Hypoxic culture was performed at 37 °C with 1% O_2_ and 5% CO_2_. Bafilomycin A1 was purchased from Sigma-Aldrich.

### Generation of Nluc-fused CD63

Plasmid vector pSpCas9(BB)-2A-Puro(PX459) V2.0 (#62988, deposited by Zhang lab) was purchased from Addgene. Construction of plasmids targeting CD63 was performed following to the protocol as per described by Zhang lab, which is available in Addgene website. The guiding oligomers used in the cloning protocol were designed as 5′-TGAGAAGATGTCAGCAATAC-3′. Upstream and downstream 1 kb each from right before the stop codon of CD63 were amplified from HCT116-derived genomic DNA, and NanoLuc gene was amplified from and pNLF1-C vector (Promega, Madison, WI, USA). Each PCR amplicon was cloned into the pUC19 vector with In-Fusion HD enzyme (Takara Bio, Shiga, Japan), and this vector was used as a donor vector. Genome editing in HCT116 was performed as follows: the day before transfection, Cells were seeded on 35-mm dish at the density of 6.25 × 10^5^ cells. The cells were co-transfected with 1 μg of cloned pX459 V2.0 and 1.5 μg of donor vector by using Lipofectamine LTX reagent (Thermo Fisher Scientific), and at 48 hours after transfection cell cloning was initiated by limiting dilution. 28 clones derived from single cells were expanded and prospective three clones were selected based on luminescence in culture medium. Finally, a clone with highest expression of CD63 (clone #17) was selected for assay. Nluc-modification of CD63 gene was confirmed by genomic PCR, sequencing and western blotting. Genomic DNA was extracted from cells by using DNeasy Blood & Tissue Kit (QIAGEN, Hilden, Germany) and genomic PCR was performed by using PrimeSTAR Max DNA polymerase (Takara Bio). Primers were as follows: human CD63, forward (F1) 5′-ctgggcaacagagcaagtct-3′, reverse (R) 5′-gaccatctcttttcggtctga-3′. PCR products were electrophoresed on a 1.0% agarose gel and visualized by staining with Syber Gold (Thermo Fisher Scientific). For sequencing, PCR product was purified by PCR purification kit (Thermo Fisher Scientific) and sequenced by human CD63 primer, forward (F2) 5′- cctgtccccatctttccttc -3′.

### NanoLuc luciferase assay

To remove the cells and cellular debris, collected culture mediums were centrifuged at 2,000 g for 10 min at 4 °C, and transferred 50 μL supernatants into white-walled 96-well plates. 50 μL Nano-Glo substrate diluted 1:50 in provided buffer (Nano-Glo Luciferase Assay System: Promega) was added, and the luciferase intensity in each well was immediately measured using an ARVO X Light luminometer (PerkinElmer, Waltham, MA, USA).

### Preparation of exosomes and Nanoparticle tracking analysis (NTA)

HT29/CD63Nluc, HCT116/CD63Nluc, HCT116/CD63Nluc-KI cells were seeded on the 150-mm culture dish at the density of 5 × 10^6^ cells, and culture for 24 hours. After washing with 20 mL phosphate-buffered saline (PBS) two times, the culture medium was replaced with 13 mL 1% exosome-depleted FBS contained medium. After 48 hours culture, to remove cells and cellular debris, the supernatant was centrifuged at 2,000 *g* for 10 min at 4 °C, and then filtered through a 0.22 μm filter (Merck, Darmstadt, Germany). To prevent aggregation of exosome, trehalose (1 M trehalose in 20 mM HEPES pH7.4) was added at the final concentration of 25 mM^52^. The supernatants were ultracentrifuged at 110,000 g for 70 min at 4 °C (SW41Ti rotor, Beckman Coulter, Brea, CA, USA), and the pellets were washed with 11 mL trehalose-contained HEPES buffer (20 mM HEPES pH7.4, 25 mM trehalose). The suspensions were re-ultracentrifuged at 110,000 g for 70 min at 4 °C, finally suspended in 200 μL HEPES buffer (20 mM HEPES, pH7.4). The size distribution and concentration of the exosomes were determined by Nanoparticle tracking analysis (NTA). NTA was performed using NanoSight LM10 instrument (Malvern Panalytical, Malvern, UK) with 488 nm laser and NTA3.1 software. Five 30 s measurements were recorded for each sample with automated analysis settings for blur, track length and minimum expected particle size. The camera level was set at 14 and the detection threshold at 10.

### Western blotting

Cells and exosomes were lysed in n-octyl-β-D-glucoside (ODG) buffer (20 mM Tris-HCl, pH7.4, 150 mM NaCl, 1 mM EDTA, 1 mM sodium orthovanadate, 20 mM NaF, 1% Nonidet P-40, 5% glycerol, 2% ODG and protease inhibitor cocktail), and immunoblotting was performed as previously described^53^. The following antibodies were used: anti-Alix (ABC40, Merck), anti-HIF1α (D1S7W, Cell Signaling Technology, Danvers, MA, USA), anti-CD63 (MX-49.129.5, Santa Cruz Biotechnology, Santa Cruz, CA, USA), anti-mCherry (1G9, MBL, Nagoya, Japan) and anti-GAPDH (6C5, Santa Cruz Biotechnology). Anti-Nluc rabbit polyclonal antibody was kindly provided by Promega.

### Biodistribution study

Long-term biodistribution of exosomes was analyzed using dorsal air sac model mice. In brief, mCherry-labeled HT29 and HCT116/CD63Nluc cells (5 × 10^6^ cells/150 μL) mixed with Matrigel were loaded into a chamber ring (Merck) covered with 0.45 μm pore size filters. The chamber rings were dorsally implanted into 5-week-old Balb/c-nu/nu female mice (Japan SLC Inc., Shizuoka, Japan). After 7 weeks of implantation, 100 μL Nano-Glo reagent diluted 1:20 in sterile PBS was intravenously and intraperitoneally injected into mice. After 3 min of administration, mice was euthanized with cervical dislocation, and organs were harvested within 7 min. Luminescence in the isolated organs were imaged with IVIS Lumina II imaging system (PerkinElmer). All animal experiments were performed under protocols approved by the Animal Care and Use Committee of Aichi Cancer Center Research Institute.

### Gene expression and shRNA

All gene transfer experiments were carried out with the pCX4 series of retroviral vectors. After Nluc gene was amplified from pNLF1-C vector (Promega) and subcloned into pCX4bsr (pCX4bsr-Nluc vector), human CD9, CD63 and CD81 were subcloned into pCX4bsr-Nluc. The production and infection of retroviral vectors were performed as described previously^54^. For gene silencing, Lentiviral vectors, both empty and carrying human Alix (ID: NM_013374.2) were purchased from Sigma.

### Soft-agar colony formation assay

Single-cell suspensions of 1 × 10^4^ cells were plated onto six-well culture dishes in 1.5 mL of DMEM containing 10% FBS and 0.36% agar on a layer of 2 mL of the medium containing 0.7% agar. 7 days after plating, colonies were stained with 3-(4,5-dimehylthiazol-2-yl)-2,5-diphenyltetrazolium bromide (MTT), and photographs of the stained colonies were taken, and number of stained colonies was counted with photoshop^55,56^.

### Cellular uptake assay

*Direct addition system*: Host cells were seeded in white-walled 96-well plates at a density of 2.5 × 10^4^ cells/well, and culture for 24 hours. Exosomes isolated from HT29/CD63Nluc and HCT116/CD63Nluc were added into each well, and the cells were incubated for 2 hours. After washing twice with PBS, they were lysed with Nano-Glo substrate diluted in provided buffer, and then luminescence was measured using ARVO X Light luminometer (PerkinElmer).

*Co-culture system*: Cellular uptake assay in co-culture condition was performed using a 24 multiwell insert system with 1.0 μm-pore PET membrane (Corning, Corning, NY, USA). As recipient cells, MEFs, A431, A549, HT29 and HCT116 cells (1 × 10^5^ cells in 1 mL medium) were seeded into the bottom chamber and engineered-donor HT29/CD63Nluc or HCT116/CD63Nluc cells (2 × 10^4^ cells in 600 μL medium) were seeded in the upper chamber. After 48 hours culture, recipient cells were washed with PBS three times, and then were lysed with Nano-Glo substrate diluted in provided buffer. The lysate was transferred to white-walled 96-well plate, and luminescence was measured using ARVO X Light luminometer (PerkinElmer).

### Statistical analysis

All summary data were reported as means ± S.D. calculated for each group and compared using the Student’s *t* test using Excel software (Microsoft). Test results were reported as two-tailed p-values, where P < 0.05 was considered statistically significant.

## Electronic supplementary material

Supplementary Methods and Figures

## Data Availability

All data generated or analyzed during this study are included in this published article.
