# Supplementary material for: Sensitive and rapid quantification of exosomes by fusing luciferase to exosome marker proteins
Source: Sci Rep. 2018 Sep 19;8:14035. doi: 10.1038/s41598-018-32535-7 (PMC6145919; doi:10.1038/s41598-018-32535-7)
Supplement: Supplementary file 1 — Supplementary Methods and Figures [file 41598_2018_32535_MOESM1_ESM.pdf]

Sensitive and rapid quantification of exosomes by fusing luciferase to exosome marker proteins

Tomoya Hikita[1], Mamiko Miyata[1], Risayo Watanabe[1] and Chitose Oneyama[1,2]*

[1] Division of Cancer Cell Regulation, Aichi Cancer Center Research Institute, Chikusa-ku, Nagoya, Japan., [2]JST, PRESTO, Nagoya, Japan

*Correspondence should be addressed to: Chitose Oneyama, Ph.D.

Division of Cancer Cell Regulation, Aichi Cancer Center Research Institute, Chikusa-ku, Nagoya 464-8681, Japan

E-mail: coneyama@aichi-cc.jp

Tel: +81-52-764-2979

Fax: +81-52-763-5233

Supplementary Methods

Immunocytochemistry

Cells cultured on collagen-coated coverslips were fixed with 4% paraformaldehyde (PFA) for 15 min, and permeabilized with 0.25% saponin/PBS for 5 min. The samples were blocked with 1% BSA/PBS for 30 min, and then incubated with primary antibodies overnight at 4°C. After incubation with Alexa488 and 564-cojugated secondary antibodies for 1h at room temperature, the coverslips were mounted on glass slides with Prolong Gold antifade reagent (Thermo Fisher Scientific). Fluorescence was observed using ZEISS LSM 800 with Airyscan confocal microscope (Carl Zwiss, Oberkochen, Germany).

Supplementary Figures

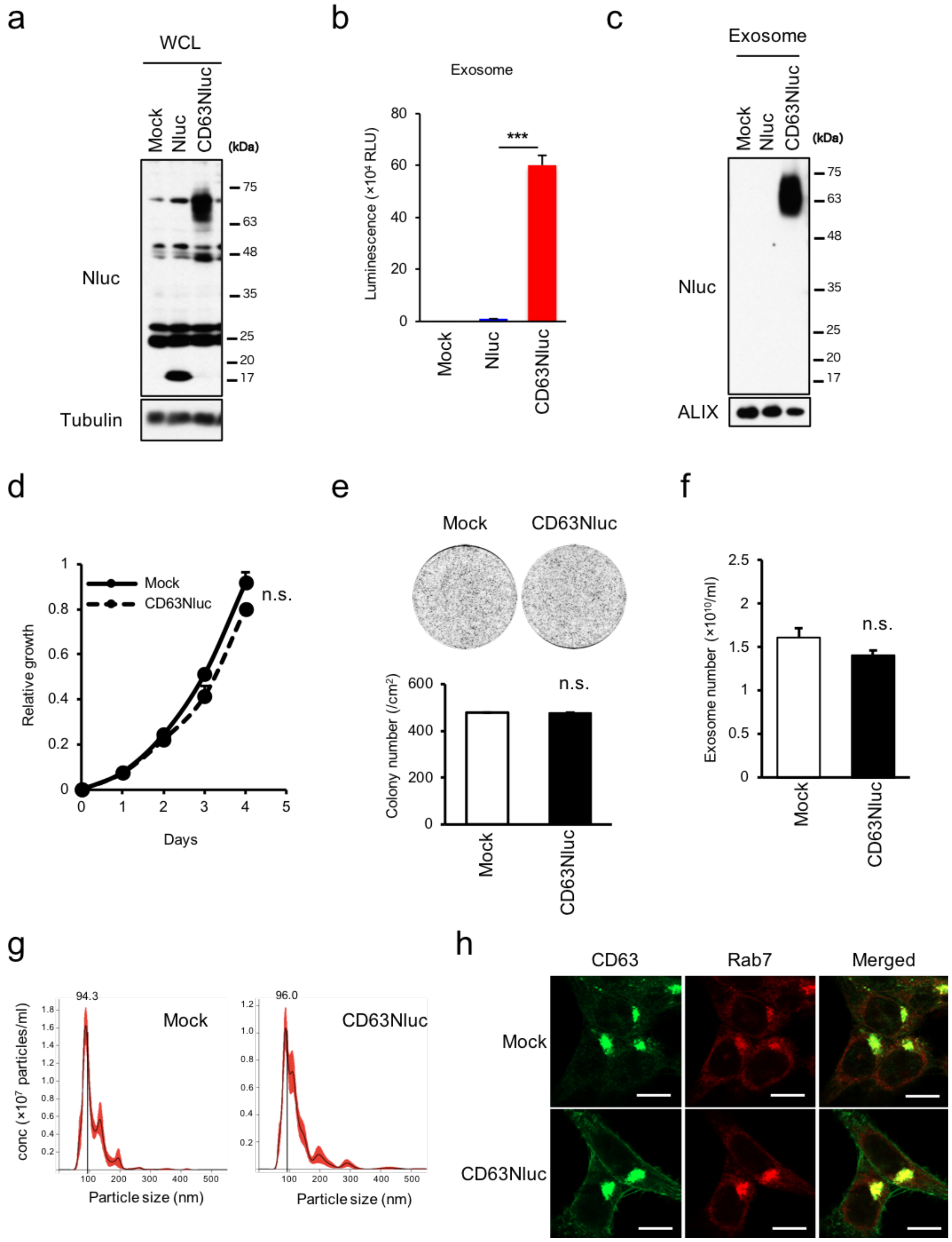

Supplementary Figure 1

(a) Western blot analysis of control (Mock), Nluc, or CD63-fused Nluc (CD63Nluc) expressing HCT116 cells. Total cell lysates from the cells were immunoblotted with the

indicated antibodies. (b) Nluc intensity of exosomes in culture medium of the cells indicated in (a). (c) Western blot analysis of Nluc expression in exosomes secreted from the cells indicated in (a). ALIX was used as an exosome marker protein. (d) Effect of ectopic Nluc-fused CD63 on anchorage-dependent growth. Growth curve is based on WST-1 assays performed on parental (Mock) and CD63Nluc-expressing HCT116 cells. (e) Effect of ectopic Nluc-Fused CD63 on anchorage-independent growth, as determined by soft-agar colony formation assay. (f) Effect of ectopic Nluc-fused CD63 expression on exosome production. (g) NTA analysis for the size distribution of isolated exosome particles. (h) The intracellular localization of CD63 in CD63Nluc expressing HCT116 cells was analyzed by immunostaining with anti-CD63 or anti-Rab7. Scale bar = 10 µm. Results are expressed as means ± SD of three wells. All data are representative of at least three-independent experiments. ***$P < 0.001$, and n.s indicates no significant difference by two-tailed Student's *t*-test.

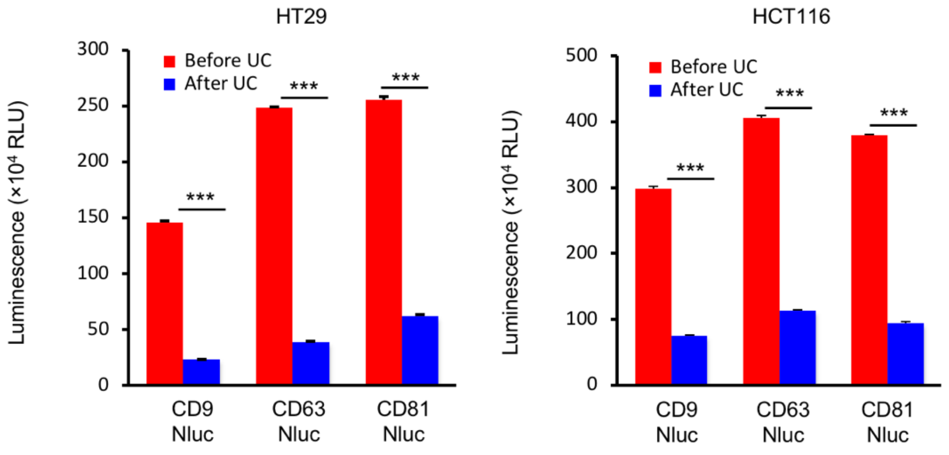

Supplementary Figure 2
Nluc intensity before and after ultracentrifugation (UC) in culture medium of CD9-, CD63-, or CD81-expressing HT29 (left panel) and HCT116 cells (right panel). Results are expressed as means ± SD of three wells. All data are representative of at least three-independent experiments. ***$P < 0.001$ by two-tailed Student's *t*-test.

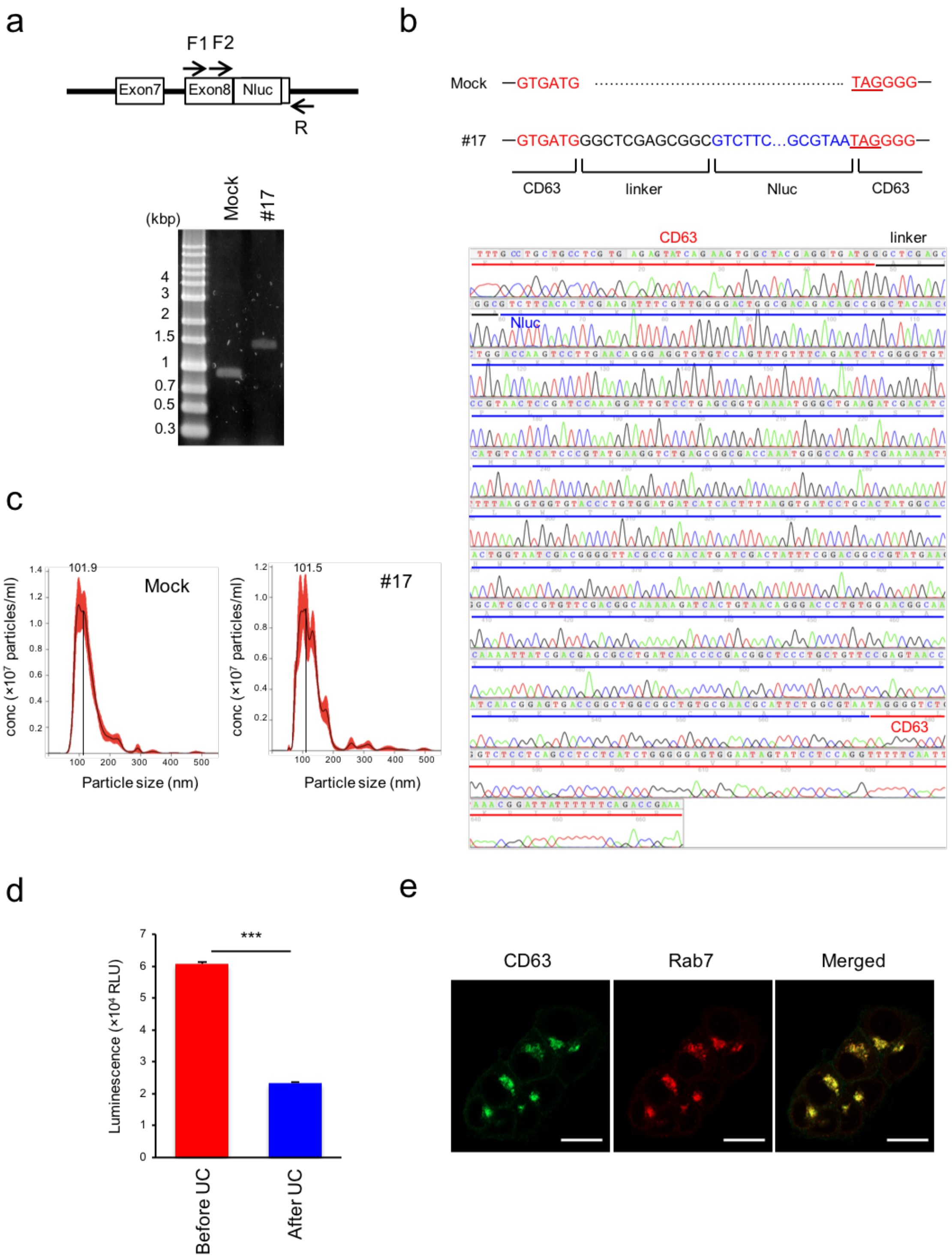

Supplementary Figure 3

(a) Gene-specific PCR with primers in the exon8-Nluc junction (F1 or F2) and 3'UTR (R) of human *CD63*. Genomic DNA was prepared from parental (Mock) and CD63Nluc knock-in (clone#17) HCT116 cells. Genomic PCR was performed with F1 and R primers and PCR products were electrophoresed on a 1.0% agarose gel and visualized by staining. (b) Sanger sequencing results of Nluc insertion at the 3' end of the *CD63*

gene using F2 primer indicated in (a). (c) NTA analysis for the size distribution of isolated exosome particles. (d) Nluc intensity in culture medium of CD63Nluc knock-in HCT116 cells before and after ultracentrifugation. (e) The intracellular localization of CD63 in CD63Nluc knock-in HCT116 cells was analyzed by immunostaining with anti-CD63 or anti-Rab7. Scale bar = 10 μm. ***P < 0.001 by two-tailed Student's *t*-test.

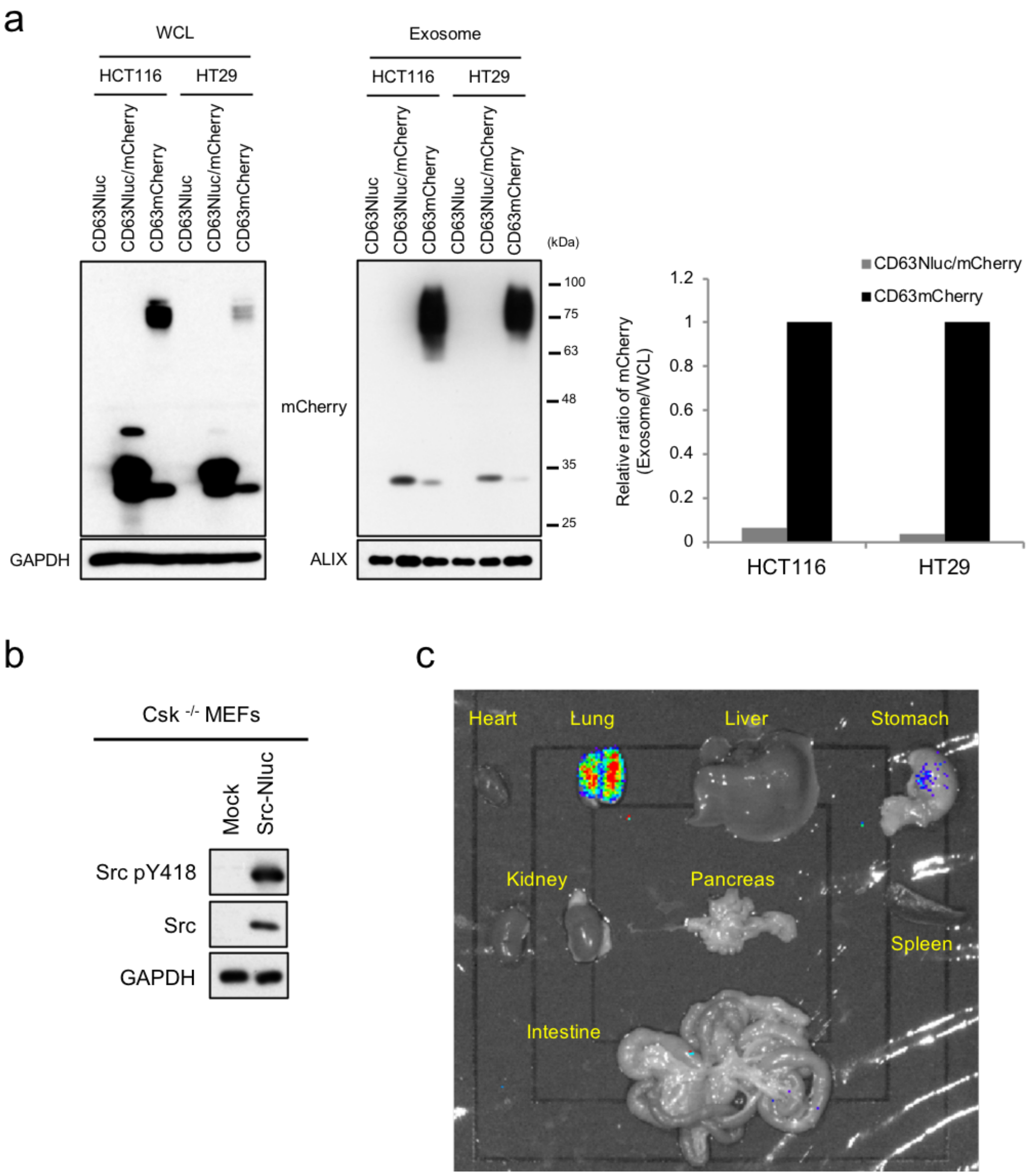

Supplementary Figure 4

(a) Western blot analysis of whole cell lysates (WCL) and exosomes from HCT116 and HT29 expressing CD63-fused Nluc (CD63Nluc), CD63-fused Nluc (CD63Nluc) and mCherry (CD63Nluc/mCherry), or CD63-fused mCherry (CD63mCherry). ALIX was used as an exosome marker protein (middle panel). Relative ratio of mCherry protein expression (Exosome/WCL) in CD63Nluc/mCherry to that of CD63mCherry was shown in graph (right panel). (b) Western blot analysis of ectopically Src-Nluc–expressing Csk-deficient mouse embryonic fibroblasts (MEFs). Total cell lysates from Src-Nluc–expressing cells were immunoblotted with the indicated antibodies. (c) Bioluminescence images in organs harvested from Src-Nluc MEF-encapsulated chamber–bearing mice. Chamber rings loaded with a mixture of Src-Nluc MEFs and Matrigel were dorsally implanted into 5-week-old Balb/c-nu/nu female mice. At 6 weeks after implantation, furimazine, a Nluc substrate, was intravenously administered, and then the luminescence in each organ was imaged by IVIS.

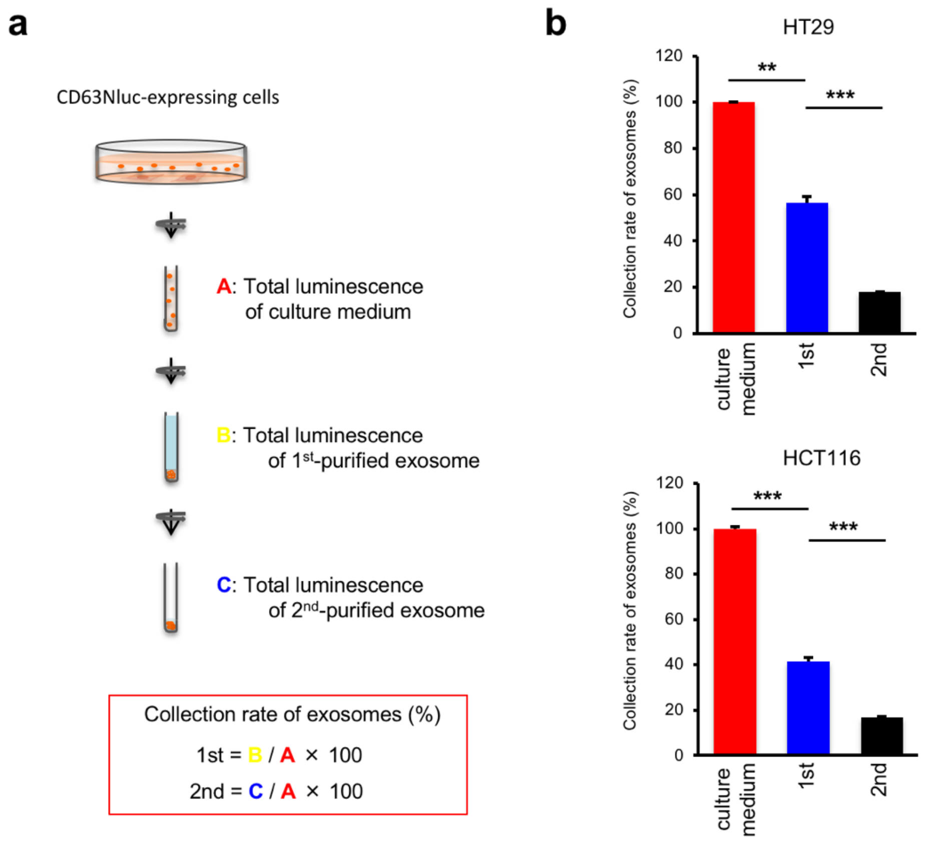

Supplementary Figure 5

(a) Schematic representation of analysis of the rate of collection of exosomes by ultracentrifugation. A, total luminescence in the culture medium after removal of cells and debris by centrifugation and filtration; B, total luminescence after the first ultracentrifugation step; C, total luminescence after the second ultracentrifugation step. Collection rates in each step were calculated according to the indicated formula, c. (b) Collection rate of exosomes in CD63Nluc-expressing HT29 (upper panel) and HCT116 (lower panel) after the first and second ultracentrifugation. $^{**}P < 0.01$ and $^{***}P < 0.001$ by two-tailed Student's *t*-test.

Figure. 1a

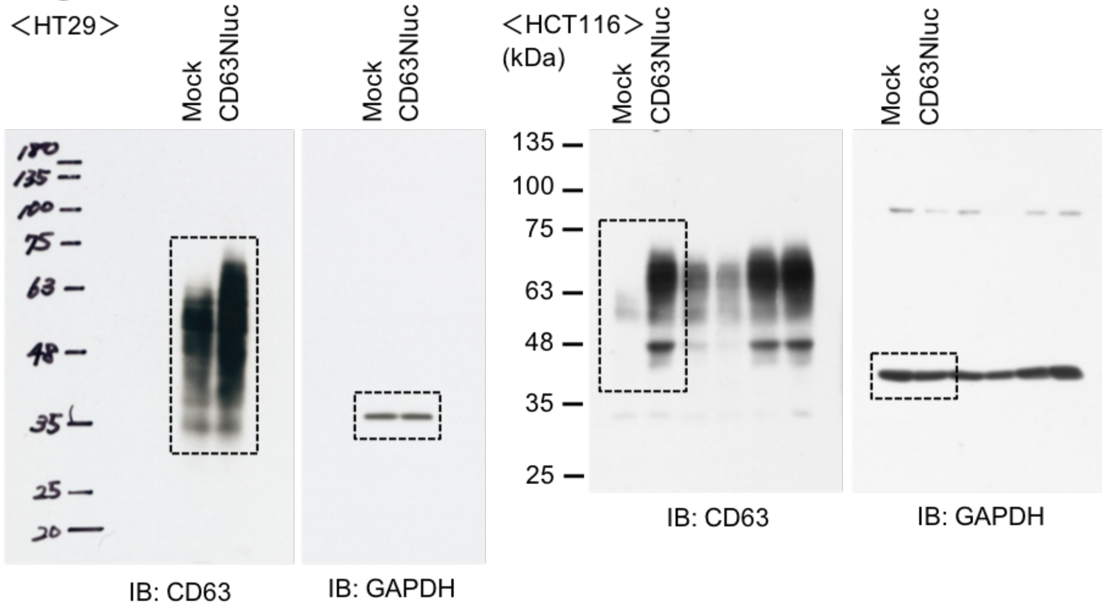

Figure. 1d

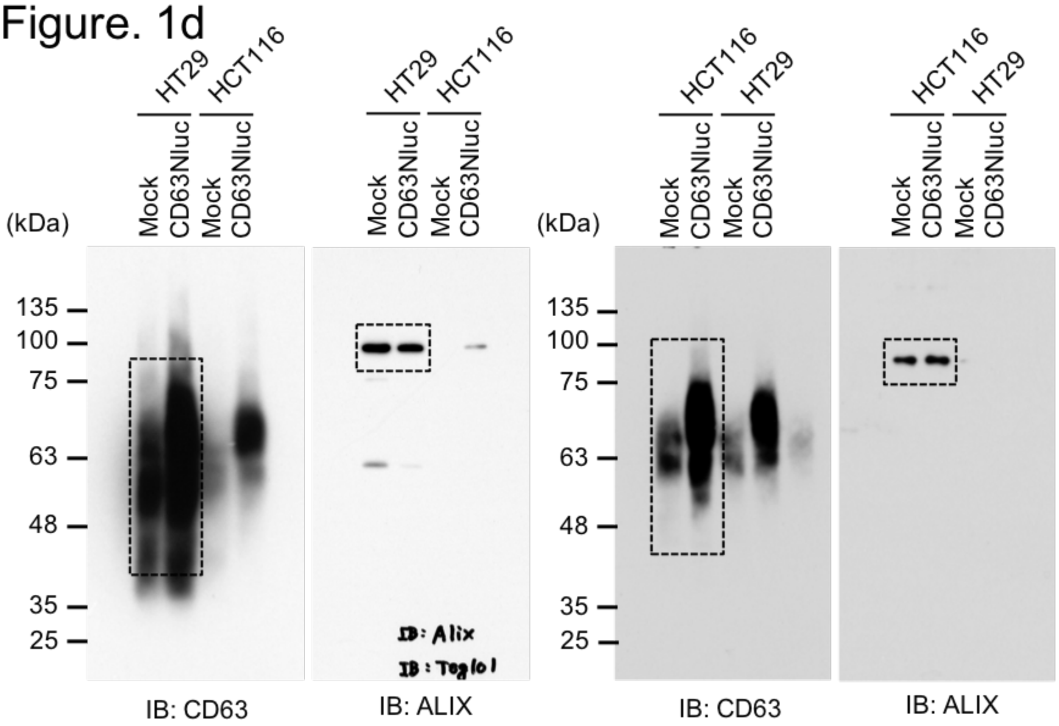

Figure. 1d

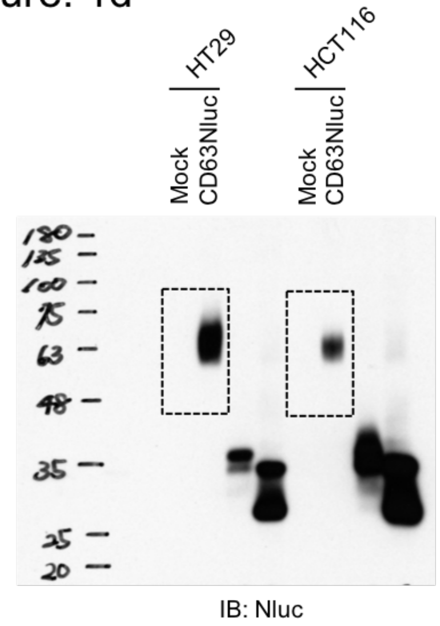

Figure. 2a

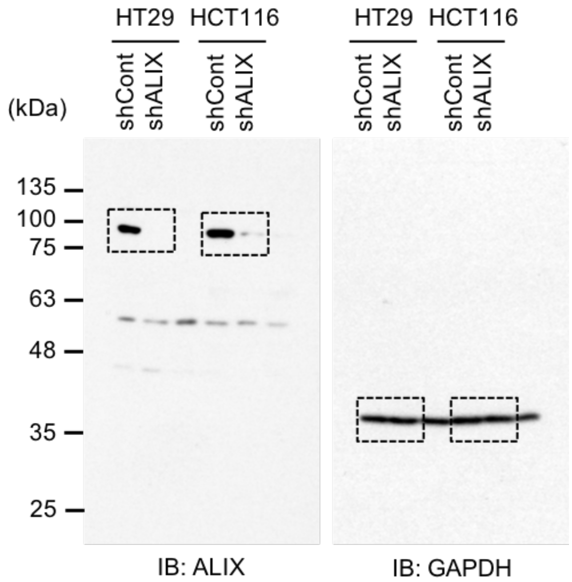

Figure. 2e

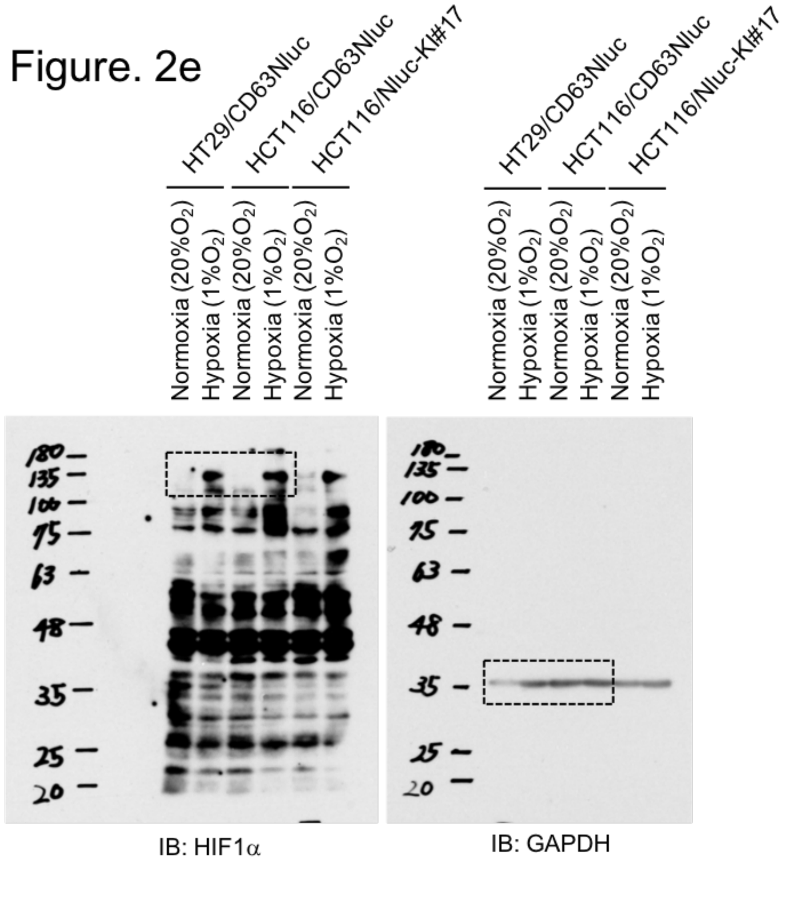

Figure. 3a

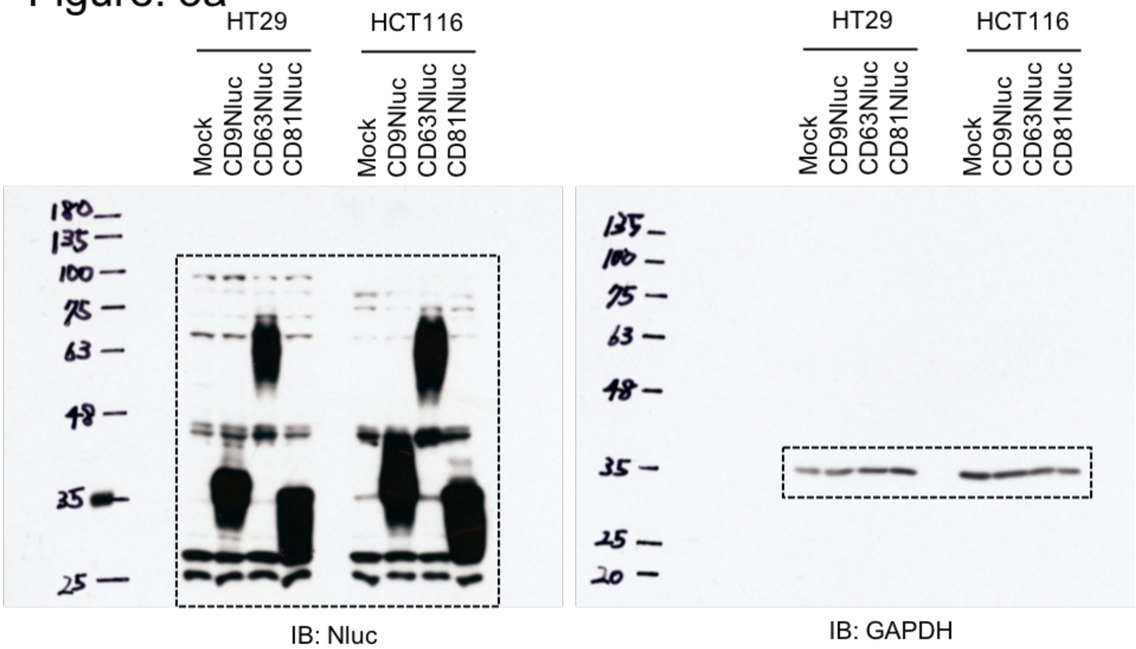

Figure. 4b

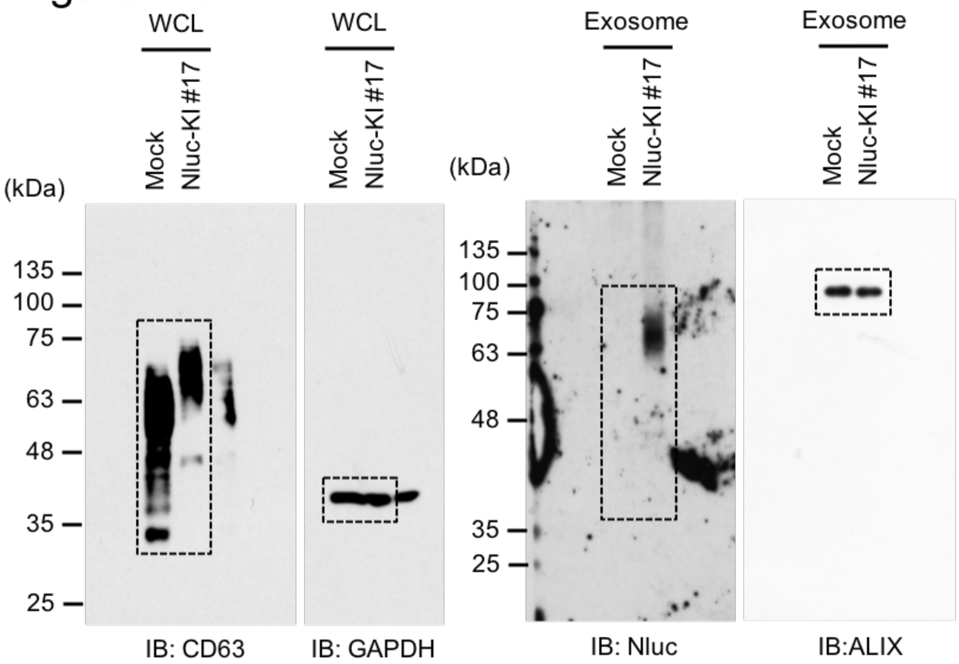

Supplementary Figure 6
Full scan images of Figures 1a, 1d, 2a, 2e, 3a, and 4b.
